# Anisotropic Photoelectric Properties of Aligned P3HT Nanowire Arrays Fabricated via Solution Blade Coating and UV-Induced Molecular Ordering

**DOI:** 10.3390/ma18112649

**Published:** 2025-06-05

**Authors:** Qianxun Gong, Jin Luo, Chen Meng, Zuhong Xiong, Sijie Zhang, Tian Yu

**Affiliations:** 1 College of Physics, Sichuan University, Chengdu 610065, China; suoketuo@163.com (Q.G.); 19150773780@163.com (J.L.); mengchen2023@stu.scu.edu.cn (C.M.); 2 College of Mechanical Engineering, Guizhou University of Engineering Science, Bijie 551700, China; zhxiong@swu.edu.cn; 3 Chongqing Key Laboratory of Micro & Nano Structure Optoelectronics, School of Physical Science and Technology, Southwest University, Chongqing 400715, China

**Keywords:** conjugated polymer, crystallization, polymer semiconductor nanowires, poly(3-hexylthiophene), light-dependent resistor

## Abstract

This paper reports on the anisotropic optoelectronic properties of aligned poly(3-hexylthiophene) (P3HT) nanowire (NW) arrays fabricated via blade coating and UV irradiation, exhibiting a remarkably high electrical resistance anisotropy ratio of up to 8.05 between the parallel (0°) and perpendicular (90°) directions. This resistance anisotropy originates from the advantage of directional charge transport. Optimized 5 mg/mL P3HT solutions under 32 min UV irradiation yielded unidirectional π-π*-stacked NWs with enhanced crystallinity. Polarized microscopy and atomic force microscopy confirmed high alignment and dense NW networks. The angular dependence of polarization exhibits a cosine-modulated response, while the angular anisotropy of the measured photocurrent points to structural alignment rather than trap-state control. The scalable fabrication and tunable anisotropy demonstrate potential for polarization-sensitive organic electronics and anisotropic logic devices.

## 1. Introduction

Organic semiconductor (OSC) materials have demonstrated broad application prospects due to their advantages in flexibility, low cost, and biocompatibility. Solution-processable conjugated polymers (CPs), with their unique optoelectronic properties, have attracted significant attention as promising alternatives to inorganic semiconductors in flexible and lightweight electronic devices [1,2,3,4,5], including light-emitting diodes [6], thin-film transistors [7,8], solar cells [9], and sensors [10,11]. However, the development of these materials has been hindered by challenges in controlling film morphology and molecular packing. The disordered molecular arrangement and insufficient crystallinity in conventional thin films severely limit charge transport efficiency, restricting their applications in electronic and optoelectronic devices [10,12]. For regioregular P3HT, a widely studied CP material, despite its excellent solution processability, charge transport characteristics, and self-assembly capability, the random orientation of π-π* stacking in polymer backbones continues to constrain its electrical performance [13,14]. Previous studies suggest that constructing densely aligned nanowire surface textures can effectively enhance carrier mobility [15]. The implementation of one-dimensional nanostructures, such as NWs or nanofibers (NFs), offers a viable solution to address phase separation caused by mixed crystalline and amorphous domains [10,12,16,17,18].

Controlling molecular alignment is critical for optimizing charge transport pathways in organic semiconductors. Current research typically employs solution processing techniques including spin-coating [19], drop-casting [20], dip-coating [21], 3D printing [22], and solution shear-coating [23,24] to regulate polymer film microstructures. Among these, solution shear coating has proven particularly effective in achieving aligned conjugated polymer NWs [24], thin films [25,26], and small molecules [27]. This technique significantly enhances charge transport properties by inducing directional alignment of semiconductor NWs within insulating matrices and such alignment directly influences the anisotropic behavior of optoelectronic devices. Ordered films exhibit remarkable anisotropic characteristics, where electrical conductivity is significantly higher along the polymer chain alignment direction compared to the perpendicular orientation. This phenomenon demonstrates that structural ordering can be strategically manipulated to control charge transport properties, providing a novel approach for developing anisotropic optoelectronic devices [24].

This study systematically investigates the properties of highly ordered one-dimensional conjugated polymer NW array films fabricated via solution blade coating technology. The blade coating method employed in this instance offers the advantage of operational simplicity. Compared to conventional shear coating it reduces the contact area between the substrate and the top cover plate, thereby decreasing material waste and improving the stability of the applied pressure. During film deposition, mechanical shear forces promote polymer backbone extension, planarization, and alignment, thereby enhancing molecular ordering and minimizing interfacial defects. Electrical characterization of films with different orientations revealed significant variations in channel conductivity. Furthermore, combining shear forces with UV-induced molecular ordering provides a synergistic route to enhancing crystallinity. Through systematic exploration of the relationship between P3HT solution preparation protocols, molecular ordering in films, and their optoelectronic properties, we further optimized the growth conditions for highly anisotropic OSC films via crystal orientation control, achieving effective one-dimensional charge transport confinement. Focusing on P3HT blade-coated films, this work combines static absorption spectroscopy, atomic force microscopy (AFM), polarized optical microscopy (POM), birefringence spectroscopy, and optoelectronic characterization to elucidate the effects of solution processing and shear-induced alignment on the molecular ordering, morphology, and charge transport of P3HT NWs. The interplay between UV irradiation time and solution concentration is revealed as a key factor governing nanowire alignment. Experimental results demonstrate that the resistive anisotropy of films can be modulated by adjusting molecular ordering levels, highlighting the potential of one-dimensional aligned NW arrays in high-performance electronic devices. Furthermore, anisotropic photocurrent responses were observed in differently oriented films, with this anisotropy shown to originate not from trap-state variations but from intrinsic structural ordering. This mechanistic insight clarifies the fundamental origin of performance anisotropy in aligned polymer systems. These findings provide new insights for developing high-performance organic–inorganic composite semiconductor materials.

## 2. Materials and Methods

### 2.1. Materials and Solution Preparation

P3HT (MW=74 kDa, >97.3% RR) was purchased from Ossila Corporation (Sheffield, UK) and used as received. A total of 35 mg of P3HT was introduced into 3.5 mL of chloroform (anhydrous grade, Chron Chemical, Chengdu, China) and heated at 55 °C for 60 min in a 20 mL glass vial fitted with a magnetic stir bar. The prepared P3HT solution was cooled to ambient temperature before UV irradiation. Subsequently, the solution was further diluted to prepare three different concentrations of P3HT precursor solutions: 10 mg/mL,5 mg/mL, and 2.5 mg/mL of 1mL and 0.5 mL of the P3HT solution were added from the vial to another two vials and 1 mL and 1.5 mL of chloroform were added to obtain 5 mg/mL and 2.5 mg/mL solutions, respectively. These P3HT precursor solutions were subjected to ultraviolet (UV) irradiation (the bottleneck covered with polyethylene film) at a wavelength of 254 nm (5 W) for varying durations ($t_{UV}$ = 0, 2, 4, 8, 16, and 32 min) to generate P3HT nanowire aggregate solutions. The aggregated P3HT solutions containing nanowires were then analyzed using UV-VIS absorption spectroscopy (Hanonlab i5, Hanon Advanced Technology Group Co., Ltd., Jinan, China) to characterize their optical properties. The concentration of solution characterization was 33.3 μg/mL and the scanning wavelength range was 300–1100 nm.

### 2.2. Fabricating the P3HT Films

The film was prepared using a blade coating technique as part of a programmable equipment setup. The glass substrate was cleaned using standard procedures and underwent surface pretreatment with chloroform. The substrate and blade moved relative to each other at a controlled speed of 4 mm/s, with a stroke length of 30 mm. A volume of 50 μL of P3HT NF aggregate solution was deposited onto the glass substrate during coating. The P3HT film was characterized using POM (Cossim POL1520, Suzhou Jingtong Instrument Co., Ltd., Suzhou, China), UV-VIS absorption spectroscopy, and a custom-built linear polarization angle measurement setup. Morphology and NF alignment order were analyzed via AFM (Bruker Dimension Icon, Billerica, MA, USA) combined with software tools.

### 2.3. Electrical Property Measurements

We fabricated P3HT films on ITO-coated glass substrates using a similar blade coating technique. The electrodes were laser-etched ITO electrodes with a sheet resistance of 10 Ω/sq and a thickness of 200 nm. The electrodes featured widths of 1500 μm and channel lengths of 100 μm, with adjacent electrodes rotated by 15° to vary the alignment direction. Transport properties of blade-coated P3HT films containing aggregated nanowires (fabricated under varying conditions) were directly characterized via direct current (DC) IV measurements (Keithley 2400, KEITHLEY, Solon, OH, USA). In addition, the photoresponse properties of the P3HT films were evaluated under 555 nm green light illumination with a constant bias voltage of 4 V applied across the electrodes, using a Keithley 2400 source meter to record the photocurrent generation dynamics.

## 3. Results and Discussion

**Figure 1:**
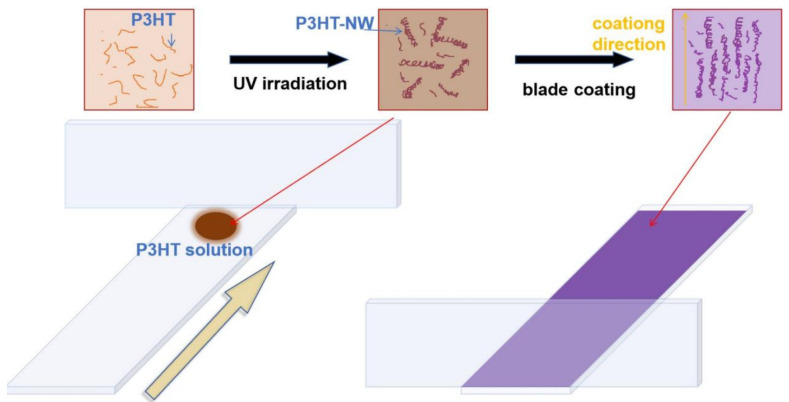
Schematic illustration of the blade coating process for thin film fabrication and growth mechanism of P3HT nanowires under UV irradiation. The yellow arrow indicates the direction of movement of the substrate glass slide.

Figure 1 illustrates the fabrication process of aligned P3HT nanowire thin films via the blade coating method. Following protocols reported in previous publications [14], a P3HT/chloroform solution was prepared in a 20 mL borosilicate glass vial and subjected to low-intensity UV irradiation (λ=254 nm). As a marginal solvent for P3HT, chloroform induces the self-assembly of P3HT chains into entangled nanowire networks. UV irradiation disrupts the twisted or entangled configurations of P3HT chains (the initial state of rr-P3HT chains is randomly coiled) which then straighten to form rod-like structures. These rod-like structures subsequently undergo π-π* stacking to form two-dimensional lamellae, and the lamellae further stack to create nanowires [28], while the fresh blend solution transitions from bright orange to dark brown (solution) upon UV exposure, indicating the formation of ordered P3HT aggregates [29]. After the solvent evaporates, the film turns dark purple.

**Figure 2:**
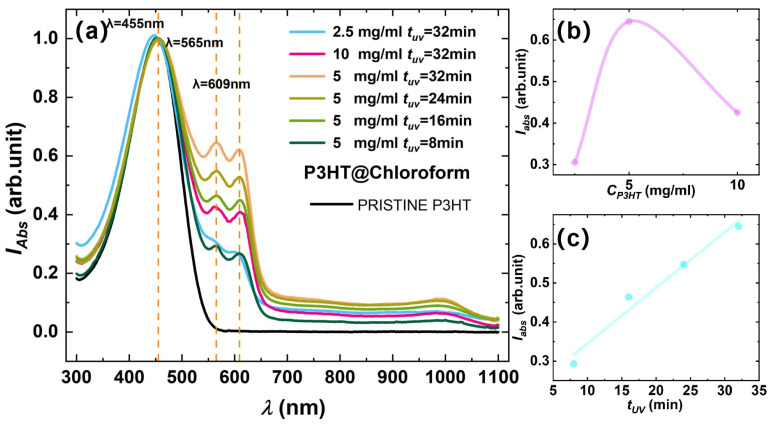
(**a**) Normalized UV-VIS absorption spectra of P3HT solution with different solution concentration and UV irradiation duration. The 3 main peaks, the absorption peaks at 455 nm, 565 nm, and 609 nm, are indicated by dashed vertical lines. (**b**) Variation in normalized peak intensity at 565 nm under different concentrations ($t_{UV}=32\;min$). (**c**) Variation in normalized peak intensity at 565 nm under different t_UV_ ($C_{P3HT}=5\;mg/mL)$.

This study utilized UV-VIS spectroscopy to investigate the regulatory role of UV irradiation on nanowire formation. Figure 2a shows the normalized dominant peak at ~455 nm in the UV-VIS absorption spectra of solutions under different irradiation conditions. The dominant peak corresponds to the intrinsic high-energy π-π* intra-band transition of P3HT. Low-energy vibronic peaks that grow at 565 nm (0–1 vibrational peak, 2.2 eV) and 609 nm (0–0 vibrational peak, 2.0 eV) are attributed to interchain exciton delocalization and coupling with vibrational modes of ordered aggregates, confirming that UV irradiation facilitates ordered assembly of P3HT chains [14,16]. The intensity ratios of these characteristic peaks relative to the main peak quantitatively reflect nanowire formation efficiency. Figure 2b demonstrates that the medium concentration solution (5 mg/mL) exhibits optimal nanowire formation efficiency. For low-concentration solutions, prolonged UV irradiation fails to generate high-intensity low-energy vibrational peaks, while excessively high-concentration solutions lead to substantial nanowire aggregation that directly forms non-solution precipitates, thereby reducing the effective solution concentration. As further revealed in Figure 2c, the P3HT solution at 5 mg/mL shows a regular enhancement in the relative intensity of the low-energy vibrational peak compared to the high-energy peak within a specific irradiation time window. This phenomenon validates that irradiation duration drives ordered assembly by strengthening π-π* interactions. Within our testing duration, P3HT nanowires did not precipitate from the solution, with the nanowire quantity displaying a quasi-linear relationship with irradiation time. Further extending UV aging time would reduce the effective solution concentration due to precipitate formation. These observations highlight the time-dependent self-assembly characteristics inherent in the system.

**Figure 3:**
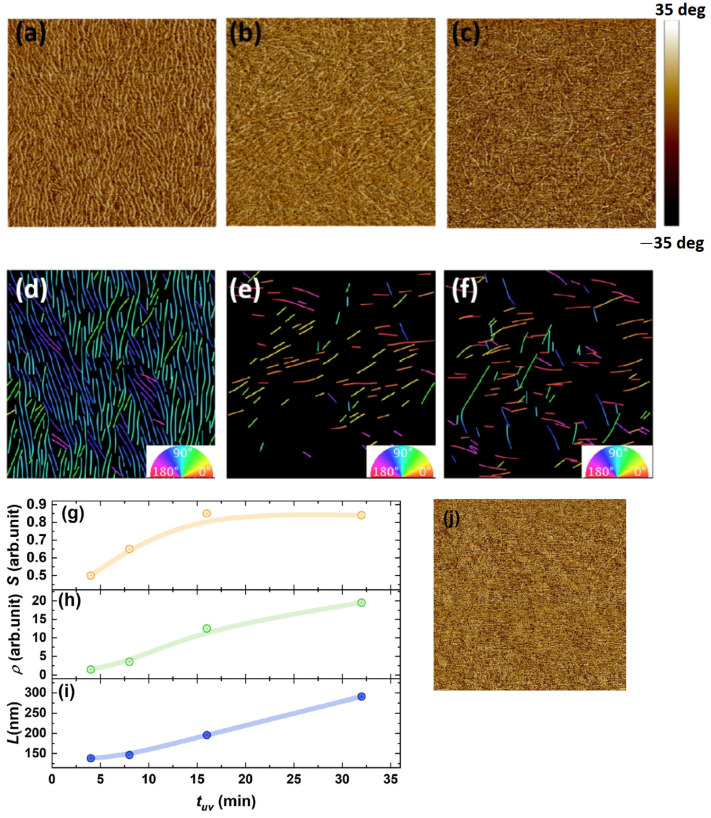
AFM images of aligned P3HT nanowire thin films under different conditions: (**a**) $t_{UV}=32\;min$, $C_{P3HT}=5\;mg/mL;$ (**b**) $t_{UV}=4\;min$, $C_{P3HT}=5\;mg/mL;$ (**c**) $t_{UV}=32\;min$, $C_{P3HT}=1.25\;mg/mL.$ The dimensions of each image are 2 μm × 2 μm. The corresponding orientation distribution data (**d**–**f**) were obtained through GT-Fiber software analysis, with the color wheel indicating orientation angles in the bottom-right insets. With $C_{P3HT}$ at 5 mg/mL under different UV irradiation times we get parameters (**g**) alignment order S; (**h**) nanowire density ρ; (**i**) average fiber length; and (**j**) AFM images of pristine P3HT film.

AFM in soft tapping mode was employed to investigate the morphological characteristics of thin films prepared under three distinct processing conditions: (a) 5 mg/mL solution with $t_{UV}=32\;min$, (b) 5 mg/mL solution with $t_{UV}=4\;min$, and (c) 1.25 mg/mL solution with $t_{UV}=32\;min$. Both (b) and (c) films exhibited nanowire formation on their surfaces, contrasting with the amorphous structure of pristine P3HT thin films [2]. However, these nanowires lacked directional alignment. In contrast, film (a) demonstrated highly crystalline, unidirectionally aligned nanowires with well-defined π-π* stacking structures. Quantitative analysis of orientational order was performed using GT-Fiber software Version 2.0 [29], generating false-color orientation maps (Figure 3d–f) that visualized the 0°–180° orientation distribution of nanostructures. The results demonstrate that under optimal concentration (5 mg/mL) and irradiation time ($t_{UV}=32\;min$), the nanowires exhibit pronounced directional alignment (Figure 3a,d). In contrast, insufficient irradiation time (Figure 3b) or low initial solution concentration (Figure 3c) leads to disordered distributions. Quantitative analysis of orientation parameters (alignment order S), nanowire density (ρ), and average length (L) for 5 mg/mL− derived films is presented in Figure 3g–i. The Herman orientation parameter (S=(⟨cos²θ⟩−0.5) quantifies anisotropy (0: isotropic; 1: anisotropic) [30]. Systematic increases were observed with prolonged $t_{UV}$. Alignment order improved from S =0.5 to 0.85, nanowire density increased ρ from 1.51 to 19.53 (a.u.) and average length L increased from 138.5 nm to 291.1 nm. S = 0.85 indicates molecular alignment approaching single-crystal levels, representing one of the higher values reported for solution-processed organic semiconductors. Our parameters, achieved without complex processing such as doping and annealing, approached those reported in previous studies (S = 0.87) [29]. These trends confirm that the UV irradiation time is a critical regulator of nanowire self-assembly. The enhancement is attributed to chloroform solvent under UV irradiation providing favorable nucleation and growth environments for P3HT molecular reorganization. The results also revealed that the ordering of nanowire alignment at $t_{UV}=16\;min$ and $t_{UV}=32\;min$ had reached relatively comparable levels, both demonstrating good alignment quality. However, both the number of nanowires and their average length continued to show progressive enhancement with extended irradiation duration.

**Figure 4:**
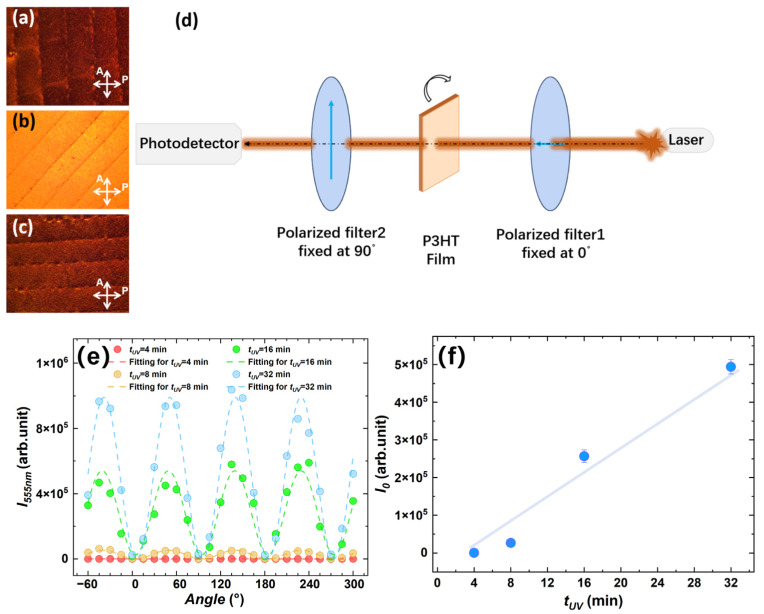
Observation of light transmission through thin films under a polarized light microscope at different angles: (**a**) 0°, (**b**) 45°, and (**c**) 90°. Pre-marked scratches on the film surface indicate directional variations. (**d**) Schematic diagram illustrating the polarization testing principle. Two mutually perpendicular polarizers were initially adjusted to extinction state. Different optical signals were detected by rotating the film sample between these polarizers. The vertical arrow and the arrow perpendicular to the paper on the two polarizers indicate their polarization directions. (**e**) Optical signal intensities of films prepared from 5 mg/mL solutions with different UV irradiation durations ($t_{UV}$ = 4 min, 8 min, 16 min, 32 min) in polarization measurements. (**f**) Averaged peak intensities under various conditions reveal that the three-polarizer system (comprising two extinction linear polarizers and the P3HT nanowire thin film polarizer) exhibits approximately linear enhancement of signal intensity with increasing solution irradiation time.

The polarized optical properties of the thin film were characterized using a POM. Figure 4a–c present the optical micrographs of P3HT NW films prepared by blade coating under cross-polarized light at different angles. Distinct birefringence textures were observed: the P3HT NW film exhibits dark textures at 0° and 90°, and bright textures at 45°, confirming the crystallinity and anisotropic morphology of the material [15,31]. Figure 4d illustrates the schematic of the optical experimental setup for quantitatively measuring angle-dependent photocurrent under green light illumination (λ=555 nm). Similar to POM principles, two orthogonal linear polarizers were positioned in the optical path to achieve extinction, and the fabricated film sample was placed between them. Rotating the sample angle altered the polarization state of the light path. Figure 4e demonstrates that the aligned P3HT film acts as a polarizer in the optical path, enabling the photodetector to receive optical signals again. The intensity follows a cosine-like angular dependence, with its amplitude showing an approximately linear correlation with UV irradiation time. This trend aligns with the evolution of nanowire density and alignment order discussed earlier. Enhanced film alignment not only depends on the orientation of nanowires but also benefits from increased nanowire density, which significantly improves polarization efficiency. Figure 4f systematically summarizes the quantitative relationship between photocurrent amplitude and UV irradiation time, revealing a similar near-linear growth trend.

**Figure 5:**
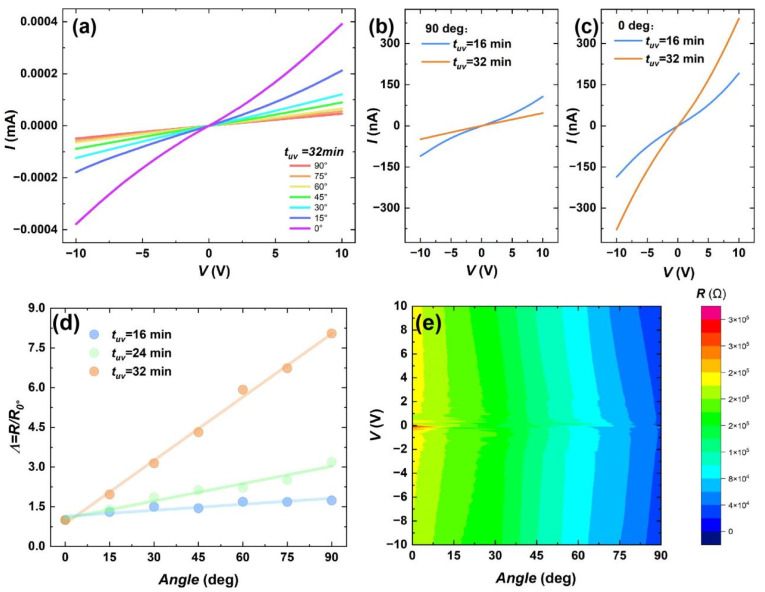
Results of in-plane current anisotropy for films prepared from a 5 mg/mL solution. (**a**) I–V characteristic curves at different angles between the applied current direction and the ordered alignment direction of nanowires in the film when $t_{UV}$ = 32 min. I–V curves under different irradiation times ($t_{UV}$ = 16 min and 32 min). (**b**) Current perpendicular to the nanowire alignment direction. (**c**) Current parallel to the nanowire alignment direction. (**d**) Normalized comparison of resistance anisotropy variations along the nanowire direction under different $t_{UV}$. (**e**) Schematic diagram showing the resistance dependence of angle and voltage at $t_{UV}$ = 32 min. The color gradient represents distinct resistance ranges, where the dependence of resistance on both voltage and angle can be simultaneously observed.

A systematic investigation was conducted on the anisotropic electrical properties of thin films. For the well-aligned sample with 32 min UV treatment and 5 mg/mL concentration, Figure 5a reveals significant resistive anisotropy between the shear direction (0°) and perpendicular direction (90°) through multiple sets of I–V curve measurements at 15° intervals, with the highest resistance ratio reaching 8.05. Compared to previous work by researchers such as Jo et al. [32] and Li et al. [33], who employed solvent vapor pretreatment to achieve 5.6-fold current enhancement and a 7.5-fold increase in anisotropy, our data demonstrates some advantages. The current amplitude exhibits a regular decreasing trend as the angle between current direction and nanowire alignment increases, demonstrating stable resistive anisotropy. Figure 5b,c show that compared with the 32 min sample, the 16 min UV-treated sample displays higher resistance in the 0° direction and lower resistance in the 90° direction, while maintaining partial alignment characteristics (anisotropy ratio: 1.74). Previous studies have confirmed that $t_{UV}$ = 16 min films already possess well-ordered nanowire alignment. This difference in anisotropy of the intensity primarily originates from nanowire density variations: the lower nanowire density in the 90° direction resulted in reduced resistance, verifying that perpendicular nanowire orientation to current flow conversely increases resistance. Additionally, both 0° and 90° directions of $t_{UV}$ = 32 min samples exhibit better I–V curve linearity compared with the $t_{UV}$ = 16 min samples in Figure 5b,c, indicating that this improved linear resistance variation arises from inherent anisotropic characteristics rather than absolute resistance values. The good symmetry of I–V curves at both coordinate axes reflects excellent film homogeneity. For samples with lower nanowire density and shorter average length, increased surface defects and inferior Ohmic contact resulted in a certain degree of rectification effect. Figure 5d presents normalized resistance–angle variation curves under three UV treatment durations, demonstrating enhanced anisotropy accompanied by nearly linear resistance–angle dependence. Voltage-dependent measurements on $t_{UV}$ = 32 min films reveal current regularity through angle variations beyond orthogonal 0°−90° directions, showing potential for continuous resistance modulation in applications. As summarized in Figure 5e, the $t_{UV}$ = 32 min film’s I–V characteristic tests indicate some nonlinear rectification effects in resistance variations, yet overall approaching homogeneous variation.

**Figure 6:**
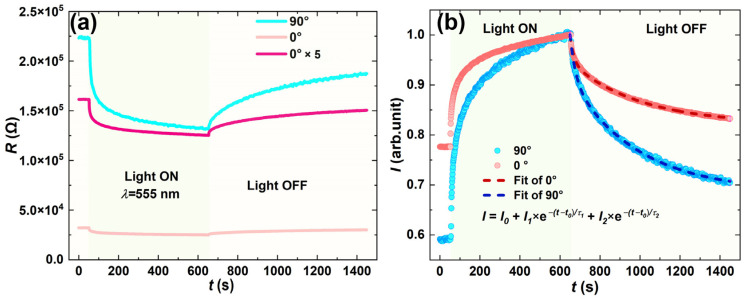
Schematic diagram of thin-film current response to light illumination: A constant voltage of V=4 V is applied in different directions. The film is irradiated with green light (λ=555nm) starting at t=50 s, and the illumination is ceased at t=650 s. (**a**) R–t (resistance–time) curves in different directions, where the data of the 0° curve is magnified 5 times for clarity. (**b**) I–t (current–time) curves normalized to the current magnitude at illumination cessation in both directions, with fitting performed on the current decay profiles.

Finally, the photoresponse characteristics of thin films under fixed bias voltage (U = 4 V) were investigated. Figure 6a displays the resistance–time curve of the sample ($t_{UV}$ = 32 min, 5 mg/mL) under green light illumination (λ = 555 nm) with illumination starting at 50 s and turning off at 650 s. The 0° and 90° samples exhibited comparable responses to light exposure. The photoresponse curve shown in Figure 6b was obtained by normalizing the post-illumination current. The data beyond 650 s were fitted to analyze the relaxation dynamics, using a double exponential function, as in Equation (1):

(1) I=I0+I1×e−(t−t0)τ1+I2×e−(t−t0)τ2

where $t_{0}=650\;s$. For the light-on phase between 50 s and 650 s, we can also obtain an approximate fit, as shown in Equation (2):

(2) I=I0′−A1×e−t−t0′τ3−A2×e−t−t0′τ4

where t_0_′ = 50 s. The two distinct attenuation mechanisms within the thin film are denoted as $\tau_{1}$ and $\tau_{2}$, while I₁ and I₂ represent their respective proportions. Both sets of experimental results are summarized in Table 1. However, since the light-on phase is influenced by light intensity and the illumination conditions cannot be guaranteed to be identical across two experiments, whereas the non-light-off phase is solely governed by the internal properties of the material, our analysis primarily focuses on the second segment.

When light is applied, the saturation trend of photocurrent originates from the instantaneous generation of photo-induced carriers causing rapid resistance decrease. This phenomenon may be related to π-π* stacking and reduced grain boundaries along the polymer chain direction in OSC materials. When electrons released from dissociated photo-excitons become trapped while holes dominate as charge carriers, enhanced current emerges in the channel [34]. During migration, these carriers encounter various recombination centers and are consumed. A dynamic equilibrium gradually forms between carrier generation and consumption, stabilizing the resistance as shown in the R–T curve. Regarding current decay after illumination cessation, Table 1 reveals two time constants (τ_1_ and τ_2_) with comparable magnitudes in both 0° and 90° oriented samples, indicating the coexistence of two distinct carrier recombination mechanisms with different (long and short) decay times. Further analysis of current component contributions is shown in Equations (3) and (4):

(3) I1,0degI1,0deg+I2,0deg=0.70

(4) I1,90degI1,90deg+I2,90deg=0.72

The nearly identical results demonstrate insignificant orientation-dependent differences in the proportional contributions of recombination mechanisms, revealing isotropic characteristics. AFM analysis reveals that in samples with low UV irradiation time ($t_{UV}$ = 4 min) or low concentration, the nanowires exhibit disordered alignment and higher defect density (Figure 3b,c). Such samples display low electrical resistance anisotropy ratios (Figure 5d, anisotropy ratio ≈ 1.74 at $t_{UV}$ = 16 min). In contrast, highly aligned samples ($t_{UV}$ = 32 min) show linear I–V curves in both 0° and 90° directions (Figure 5b,c), indicating uniform electrode contacts and demonstrating that grain boundary effects do not dominate angular dependence. Although defects and grain boundaries are universally present in thin films (particularly in low-orderliness samples), AFM and electrical characterizations confirm that their spatial distribution exhibits no angular dependence. Consequently, the contribution of defects to photocurrent anisotropy can be regarded as isotropic background noise. The highly comparable decay constants (τ_1_ ≈ 350 s, τ_2_ ≈ 20 s) and current component ratios (I_1_/I_2_ ≈ 0.7) observed in the 0° and 90° directions demonstrate that the carrier recombination mechanism is orientation independent. Should trap-state distribution exhibit angular dependence, it would manifest as variations in τ values or I_1_/I_2_ ratios across directions (e.g., shortened τ in the 90° direction due to increased grain boundaries). However, experimental data do not support this hypothesis. The angle-independent nature of photocurrent decay kinetics (Table 1) indicates no significant differences in trap-state density or carrier recombination pathways between parallel (0°) and perpendicular (90°) orientations. This stands in sharp contrast to the structural alignment-dominated anisotropy—where both electrical resistance (Figure 5a) and polarized light response (Figure 4e) exhibit systematic angular variations directly correlated with nanowire density and alignment degree (Figure 3g–i). While defects and grain boundaries may reduce the overall conductivity of thin films (e.g., lower resistance in the 90° direction for $t_{UV}$ = 16 min samples compared to highly ordered samples, Figure 5c), their contribution to anisotropy is substantially weaker than that of structural alignment. The directional nanowire network serves as the core factor modulating angular-dependent resistance/photocurrent by providing continuous charge transport pathways in the 0° direction while enhancing carrier scattering at nanowire boundaries in the 90° direction.

The observed anisotropic resistance variations primarily stem from the directional alignment of nanowires themselves, enabling more stable light-responsive resistance modulation. Therefore, the anisotropy in ordered thin films shows great potential for tunable resistance and polarized light response applications.

## 4. Conclusions

This study demonstrates a scalable approach to fabricating highly aligned poly(3-hexylthiophene) (P3HT) nanowire (NW) arrays via solution blade coating coupled with UV-induced molecular ordering. Systematic optimization of P3HT concentration (5 mg/mL) and UV irradiation duration (32 min) yielded unidirectionally oriented NWs with enhanced crystallinity, high alignment order (S = 0.85), and dense packing (density ρ = 19.53 a.u.). Key findings include anisotropic charge transport, where electrical characterization revealed a remarkably high resistance anisotropy ratio of 8.05 between parallel (0°) and perpendicular (90°) orientations, attributed to directional charge transport along the π-π*-stacked NWs; polarization-dependent optoelectronics, in which the angular photocurrent response followed a cosine-modulated relationship while polarized optical microscopy confirmed birefringence and crystallinity, enabling polarization-sensitive light modulation; mechanistic insight showing that anisotropy in optoelectronic properties originates from structural alignment rather than trap-state variations, evidenced by angle-independent carrier recombination dynamics (τ_1_ ≈ 350 s, τ_2_ ≈ 20 s) and symmetric defect distributions; and scalable fabrication, where the blade coating technique reduced material waste and improved alignment stability, offering a practical route for large-area organic electronics. These results highlight the potential of aligned P3HT NW arrays in polarization-sensitive photodetectors and anisotropic logic circuits, where tunable resistance and angle-dependent photoresponses enable novel device functionalities. Future work should explore solvent engineering to suppress NW aggregation at high concentrations (>10 mg/mL) and extend this alignment strategy to other conjugated polymers.

## Figures and Tables

**Table 1:** Summary of fitting parameters for the current decay curve in Figure 6b.

Light Off	I_0_ (a.u.)	I_1_ (a.u.)	τ_1_ (s)	I_2_ (a.u.)	τ_2_ (s)
0°	0.821	0.124	351.2	0.054	18.7
90°	0.686	0.226	339.1	0.089	27.8
Light on	I_0_′ (a.u.)	A_1_ (a.u.)	τ_3_ (a.u.)	A_2_ (a.u.)	τ_4_ (a.u.)
0°	1.00	0.111	196.1	0.105	10.9
90°	1.00	0.215	245.7	0.236	16.1

## Data Availability

The original contributions presented in this study are included in the article. Further inquiries can be directed to the corresponding author.

## Abbreviations

The following abbreviations are used in this manuscript:

P3HT	Poly(3-hexylthiophene)
NW	Nanowire
NF	Nanofiber
OSC	Organic semiconductor
CPs	Conjugated polymers
AFM	Atomic force microscopy
POM	Polarized optical microscopy
UV	Ultraviolet
DC	Direct current
